# A systematic framework to derive *N*-glycan biosynthesis process and the automated construction of glycosylation networks

**DOI:** 10.1186/s12859-016-1094-6

**Published:** 2016-07-25

**Authors:** Wenpin Hou, Yushan Qiu, Nobuyuki Hashimoto, Wai-Ki Ching, Kiyoko F. Aoki-Kinoshita

**Affiliations:** 1 Department of Mathematics, The University of Hong Kong, Hong Kong, 999077 China; 2 Faculty of Science and Engineering, Soka University, Tokyo, 192–8577 Japan; 3 Hematology Oncology Division, Northwestern University, Evanston, IL 60208 USA

**Keywords:** *N*-glycan, Glycosylation reaction networks construction, Glycosylation enzyme activity, Mass spectrum, Glycobiology

## Abstract

**Background:**

Abnormalities in glycan biosynthesis have been conclusively related to various diseases, whereas the complexity of the glycosylation process has impeded the quantitative analysis of biochemical experimental data for the identification of glycoforms contributing to disease. To overcome this limitation, the automatic construction of glycosylation reaction networks *in silico* is a critical step.

**Results:**

In this paper, a framework K2014 is developed to automatically construct *N*-glycosylation networks in MATLAB with the involvement of the 27 most-known enzyme reaction rules of 22 enzymes, as an extension of previous model KB2005. A toolbox named Glycosylation Network Analysis Toolbox (GNAT) is applied to define network properties systematically, including linkages, stereochemical specificity and reaction conditions of enzymes. Our network shows a strong ability to predict a wider range of glycans produced by the enzymes encountered in the Golgi Apparatus in human cell expression systems.

**Conclusions:**

Our results demonstrate a better understanding of the underlying glycosylation process and the potential of systems glycobiology tools for analyzing conventional biochemical or mass spectrometry-based experimental data quantitatively in a more realistic and practical way.

## Background

Glycosylation is an important and highly complex post-translational modification that generates an extensive functional capability from a limited set of genes and encompasses the biosynthesis of sugar moieties in the endoplasmic reticulum (ER) and Golgi apparatus [1–3]. Glycans are highly variable and structurally diverse compounds consisting of a large number of monosaccharides, including mannose, fucose, and galactose, linked through an enzymatic process called glycosylation [4]. Unlike protein structures, glycan structures are neither directly encoded in the genome nor arranged in a simple linear chain [5]. Instead, the structure of secreted and membrane-bound glycans is determined during their assembly in the endoplasmic reticulum and the Golgi apparatus by a controlled sequence of glycosyltransferase and glycosidase processing reactions [6].

One of the major types of glycans attached to asparagine residues of proteins, *N*-linked glycans, is determined by a manageable number of enzymes that catalyze monosaccharide attachment. *N*-linked glycosylation occurs co-translationally in endoplasmic reticulum compartments. Glycoproteins migrate into the Golgi apparatus once the protein finishes folding and some residues in the glycan trim successfully [7]. Many of these enzymes can generally accept several *N*-linked glycans as substrates, therefore generating a large number of glycan products and their glycosylation pathways [7]. Processing involves the removal of mannose groups, which is facilitated by mannosidases, and the addition of diverse monosaccharides driven by specific glycosyltransferases to the substrate glycan. Therefore, the glycosylation pathways of *N*-linked glycans comprise consecutive enzymatic steps, which are determined by the glycan structures produced by the previous enzyme, to produce a new glycan structure as the substrate of the next glycosylation reaction [7].

From research conducted in the past decades, it is clear that the glycosylation of diseased cells and healthy cells often results in different glycan changes that contribute to pathological progression, leading to the possibility that disease-specific glycan structures exist [8–12]. This has potential medical applications; for example, the potential to distinguish benign forms of prostate cancer from highly malignant cancer based on the changes in enzymes’ activities and intracellular processing events [13, 14]. Effective engineering of glycosylation pathways can potentially lead to an improved therapeutic performance of glycoprotein products. Considerable progress has been made in Prostate-Specific Antigen (PSA) research; and analytic approaches in analyzing large data sets have also been utilized to expand analyses from PSA to numerous cancers and diseases known to have abnormalities in glycosylation. However, more research is still needed in acquiring and interpreting datasets to completely characterize glycosylation, including enzymatic profiles involved in glycosylation and the large quantities of glycans produced by enzymes.

Fortunately, a wealth of data is available from glycosylation-specific databases such as the Consortium for Functional Glycomics (CFG) website [15] and GlycomeDB [16]. Also, Liquid Chromatography (LC) and Mass Spectrometric (MS) techniques have been emerging as enabling and important techniques in glycomics. A number of LC/MS methods have been incorporated into glycomics workflows for permethylated and aminated glycans reduction [17, 18]. Statistical methods have been proposed to predict glycan structures from gene expression data [19–21]. However, these traditional and qualitative methods in biochemistry or cell biology research do not provide a detailed understanding of the complex glycosylation mechanism quantitatively.

Systems biology-based mathematical models have been developed to overcome this limitation [6, 22–26]. In this regard, the construction of glycosylation reaction networks *in silico* is an important step that can enable the quantitative analysis of biochemical experimental data. Whereas several studies have been done to construct glycosylation reaction networks automatically on computers, they are limited by the lack of a systematic definition of the linkage, stereochemical specificity and reaction conditions of enzymes that are involved in the reactions.

Liu and Neelamegham [27] made a significant contribution by designing an open-source MATLAB-based toolbox, Glycosylation Network Analysis Toolbox (GNAT), for studies of systems glycobiology. This toolbox enables a streamlined machine-readable definition for the glycosylation enzyme class and the construction as well as adjustment of glycosylation reaction networks [27].

This paper extends Liu and Neelamegham’s work [27] to predict a wider range of glycans produced by enzymes encountered in human cell expression systems. In addition, our model can be applied to a larger range of experimental conditions that might be encountered in a cell culture environment. We expand the scope by inclusion of additional enzyme classes involved in gene expression data. We extend the framework of KB2005 [6] through involving 22 enzymes (27 enzyme reaction rules) in our network.

To the best of our knowledge, these 22 enzymes (27 enzyme reaction rules) are all enzymes associated with *N*-glycan that exist in Golgi compartments. Networks constructed can be used to relate the observed mass spectrometric measurements to the underlying gene expression weights. This relationship can be used to quantitatively understand how changes in enzymes’ activities affect the profile of glycan structures produced in the biosynthesis process.

## Methods

We apply the Glycosylation Network Analysis Toolbox (GNAT) as the platform to facilitate the modeling. Generated by Liu and Neelamegham [27], GNAT is a MATLAB-based toolbox for the automated construction of glycosylation reaction networks.

### Enzyme definitions using biological experimental data

To analyze biological experimental data in the glycomics field quantitatively, the construction of glycosylation reaction networks, which can describe the glycan biosynthesis process *in silico*, is rather critical. Some efforts have been made to build systems-level cellular glycosylation reaction networks on *O*-linked glycan [26] or *N*-linked glycan [23] formation. However, this work has always been hindered by the lack of a complete system for the specificity of detailed glycosylation rules. In this paper, we address this problem by defining enzymes in a machine-friendly way using a MATLAB-based toolbox called Glycosylation Network Analysis Toolbox (GNAT).

In GNAT, we can define generic enzymes using the *Enz* class. Since the properties of transferases and hydrolases are very different, *TfEnz* and *HlEnz* are used as subclasses of the *Enz* class to define them, respectively, as follows:

Transferase reaction:

$$donor+acceptor \leftrightharpoons donorProd + acceptorProd $$

Hydrolase reaction:

$$substrate + H_{2}O \leftrightharpoons prod\_oh +prod\_h $$

where *donor*, *donorProd*, *acceptor* and *acceptorProd* are variables used to specify properties in transferase (*TfEnz*) reactions and *prod*_*oh* and *prod*_*h* are for hydrolase (*HlEnz*) reactions as named.

In addition, variables such as *resfuncgroup*, *linkFG*, *resAtt*2*FG* and *linkAtt*2*FG* are used to specify functional groups and linkage specificity for enzymes. More specifically, *resfuncgroup* is the residue of functional group transferred, *resAtt*2*FG* is the residue attaching to functional group, *linkFG* refers to the linkage between attaching residue and functional group and *linkresAtt*2*FG* stands for the linkage between the attaching residue and its next neighboring residue.

**Fig. 1:**
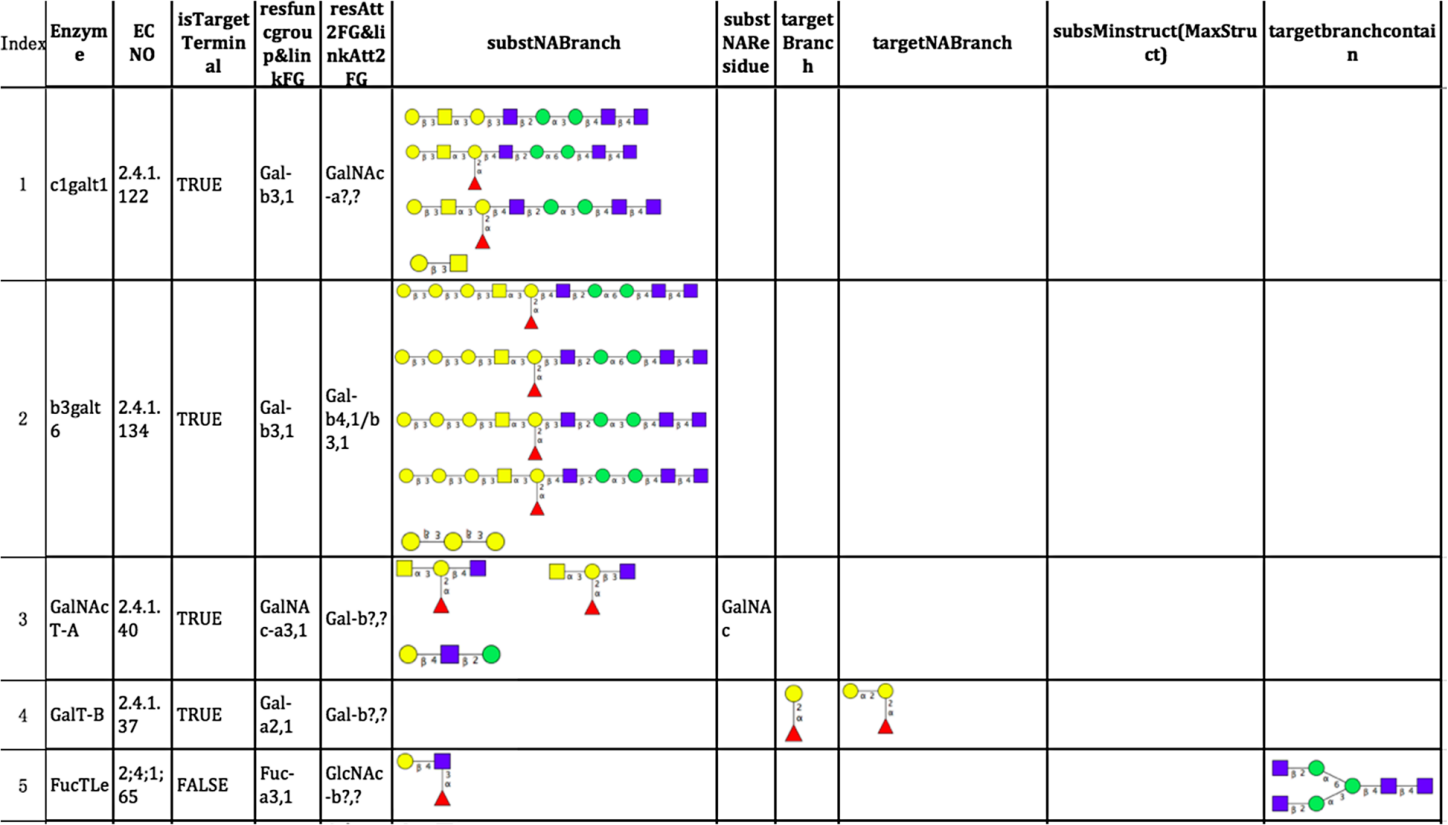
Part of enzyme reaction rules defined in class *G* *T* *E* *n* *z*/*G* *H* *E* *n* *z* (part 1). Blank fields indicate that we do not need to define the value of this variable of the corresponding enzyme

**Fig. 2:**
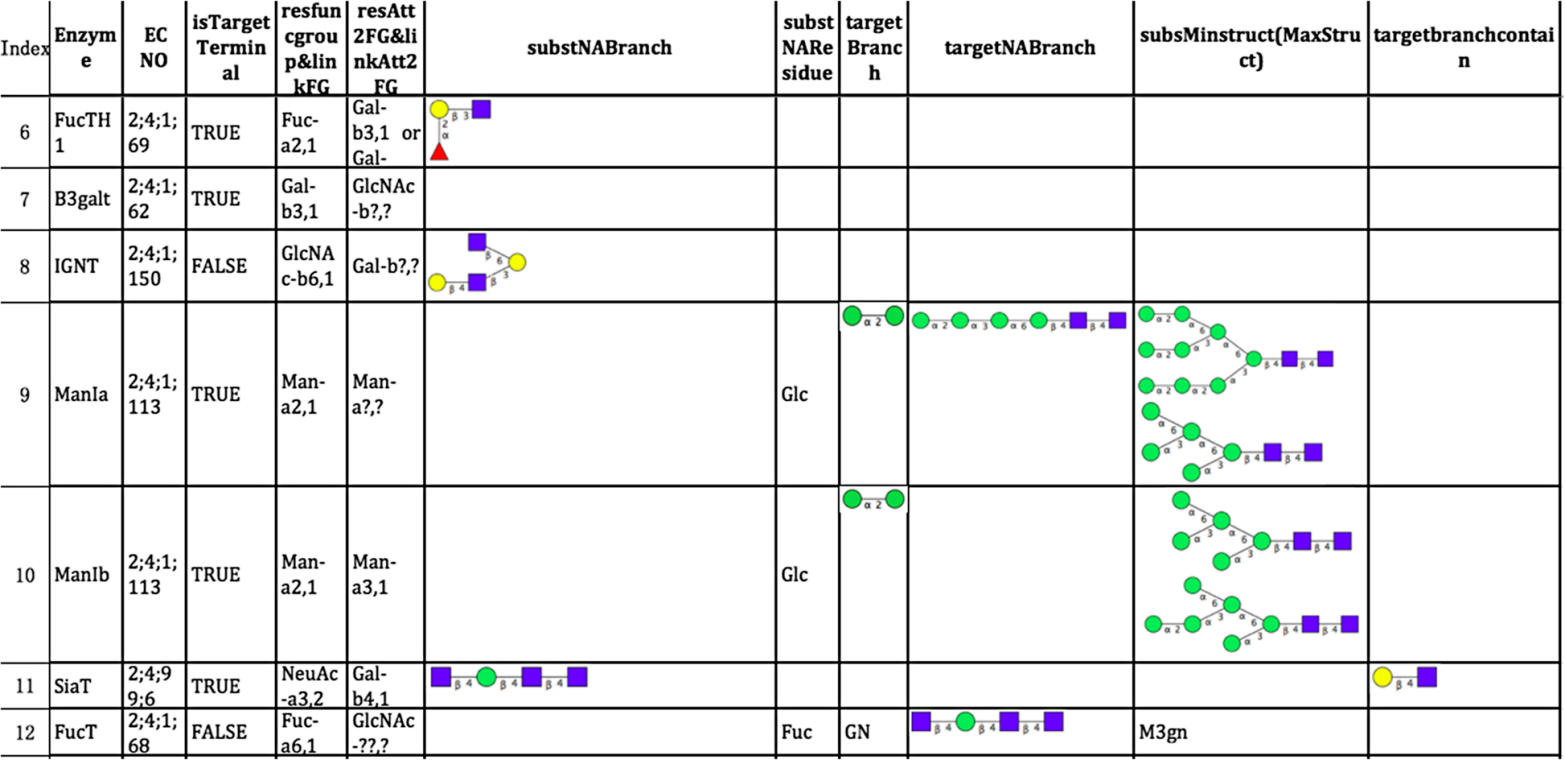
Part of enzyme reaction rules defined in class *G* *T* *E* *n* *z*/*G* *H* *E* *n* *z* (part 2). Blank fields indicate that we do not need to define the value of this variable of the corresponding enzyme

Most importantly, eight substrate specificity functions are available in the glycosyltransferase and glycosidase classes: *substMinStruct*, *substMaxStruct*, *substNABranch*, *substNAStruct*, *substNAResidue*, *targetBranch*, *targetNABranch*, and *isTerminalTarget*. They define the properties of substrates or products in reactions upon which a specific enzyme acts. Only if all conditions defined by these functions are satisfied can the corresponding enzyme act on the substrate; that is, the reaction will happen. In this work, firstly, we collect 27 enzyme reaction rules of 22 enzymes from databases, such as GlycomeDB [16], KEGG Glycan, IUBMB (International Union of Biochemistry and Molecular Biology), GlycoGene DataBase (ggdb), the Consortium for Functional Glycomics (CFG) website [25], and previous work by Sandra V. Bennun et al. [24] (See Table 1). Secondly, we define enzymes by specifying their properties in biosynthesis reactions utilizing the functions above (See Figs. 1 and 2).

**Table 1:** Current enzyme reaction rules

Index	Enzyme	EC No.	Substrate	Product	Constraint
1	ManI	[3.2.1.113]	(Ma2Ma	(Ma	$\thicksim $*2Ma3(...Ma6)
2	ManI	[3.2.1.113]	(Ma3(Ma2Ma3(Ma6)Ma6)	(Ma3(Ma3(Ma6)Ma6)	
3	ManII	[3.2.1.114]	(Ma3(Ma6)Ma6	(Ma6Ma6	(GNb2 |Ma3 & $\thicksim $Gnbis)
4	ManII	[3.2.1.114]	(Ma6Ma6	(Ma6	(GNb2 |Ma3 & $\thicksim $Gnbis)
5	FUT8	[2.4.1.68]	GNb4GN	GNb4(Fa6)GN	GNb2 |Ma3 & $\thicksim $Gnbis & $\thicksim $Ab
6	MGAT1	[2.4.1.101]	(Ma33(Ma3(Ma6)Ma6)Mb4	(GNb2Ma3(Ma3(Ma6)Ma6)Mb4	
7	MGAT2	[2.4.1.143]	(GNb2|Ma3(Ma6)Mb4	(GNb2 |Ma3(GNb2Ma6)Mb4	
8	MGAT3	[2.4.1.144]	GNb2 |Ma3	GNb2 |Ma3(GNb4)	$\thicksim $Ab & $\thicksim $Gnbis
9	MGAT4	[2.4.1.145]	(GNb2Ma3	(GNb2(GNb4)Ma3	$\thicksim $Gnbis
10	MGAT5	[2.4.1.155]	(GNb2Ma6	(GNb2(GNb6)Ma6	$\thicksim $Gnbis
11	iGnT	[2.4.1.149]	(Ab4GN	(GNb3Ab4GN	$\thicksim $_Ma3 |Mb4
12	b4GalT	[2.4.1.38]	(GN	(Ab4GN	($\thicksim $*GNb4)(…Ma6)Mb4
13	a3SiaT	[2.4.99.6]	(Ab4GN	(NNa3Ab4GN	
14	IGNT	[2.4.1.150]	(Ab4GNb3Ab	(Ab4GNb3(GNb6)Ab	
15	a6SiaT	[2.4.99.1]	(Ab4GN	(NNa6Ab4GN	
16	b3GalT1	[2.4.1.62]	(GN	(Ab3GN	($\thicksim $*GNb4)(…Ma6)Mb4
17	FUT3	[2.4.1.65]	Ab3GNb	Ab3(Fa4)GNb	(Ab3* or (Fa2Ab3* or (NNa3Ab3*)
18	FUT3	[2.4.1.65]	(…Ab4GNb	(Fa3(…Ab4)GNb	(*Ab4 or (*Fa2Ab4 or (*NNa3Ab4)
19	FUT1	[2.4.1.69]	(Ab3GNb	(Fa2Ab3GNb	
20	FUT1	[2.4.1.69]	(Ab4GNb	(Fa2Ab4GNb	
21	a3FucT	[2.4.1.152]	(…Ab4GNb	(Fa3(…Ab4)GNb	(*Ab4 or (*Fa2Ab4
22	GalNAcT-A	[2.4.1.40]	(Fa2Ab	(Fa2(ANa3)Ab	
23	GalT-B	[2.4.1.37]	(Fa2Ab	(Fa2(Aa3)Ab	
24	b3GALT6	[2.4.1.134]	Ab4GN	Ab3Ab4GN	
25	b3GALT6	[2.4.1.134]	Ab4A	Ab3Ab4A	
26	c1GALT1	[2.4.1.122]	AN	Ab3AN	
27	st3galI	[2.4.99.4]	(Ab3GN	(NNa3Ab4GN	

### Automated construction of glycosylation reaction networks

We integrate glycosyltransferase and glycosidase enzymatic data from databases into across-the-board enzyme classes: *GHEnz* and *GTEnz*. Automated construction of glycosylation reaction networks is enabled by the definition of the glycosidase (*GHEnz*) and glycosyltransferase (*GTEnz*) classes. If the substrate and the enzyme are determined, the product of glycosidase and glycosyltransferse reactions can be automatically generated by function *product inference*. Here we choose the function *forward network inference*, which can consider the products generated as the substrates of the next reaction so that reactions will happen in a sequential manner if the reaction conditions conform to the enzyme reaction rules. This is repeated until no additional new products are generated by the set of enzymes specified.

In this work, we define all enzymes in file *createEnzDb.m* (See “Availability of data and materials” section for the MATLAB codes) and then load it in MATLAB. Next, we place all enzymes in a CellArrayList object called *enzArray*. Starting with the glycan *CellArrayList* objects 9-mannose and 8-mannose as substrates, all possible reaction pathways and products are determined by function *inferGlyForwPath*. This process will continue indefinitely if there are always reactions and products. In this process, duplicate reactions and glycan product species are removed while being consolidated into the *Pathway* object. In the network generated by our model (called K2014), nodes represent the glycan species and the biochemical reactions between two glycans are denoted as edges. Setting the value of the variable *count*, we can easily limit the size of the network generated as needed.

## Results

**Fig. 3:**
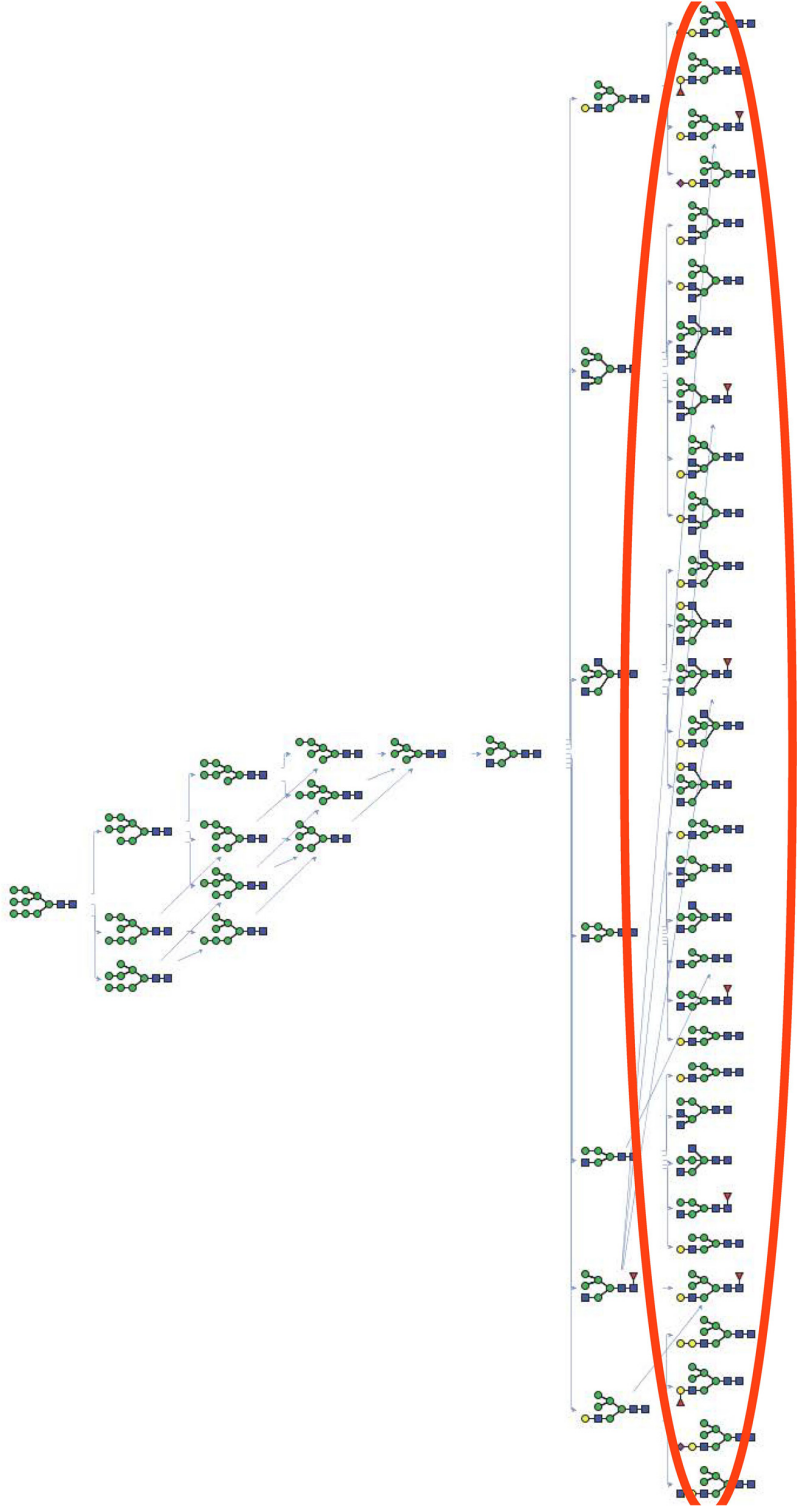
Reaction network generated by model K2014. This is the glycosylation reaction network automatically generated by our model K2014 which takes M9 as the substrate and involves all 27 enzyme reaction rules (only the first seven rounds are shown here)

We applied the 27 current reaction rules of biosynthesis reactions catalyzed by 22 enzymes to define all these enzymes. These definitions were then used to infer an *N*-linked glycosylation reaction network automatically in MATLAB. The network generated shows all enzymes catalyzing each reaction in different colors. All reactions are denoted as arrows, which are determined by the enzyme that catalyzed them. Glycan structures involving linkages and monosaccharides that constitute the glycan are also visual. We call this network K2014, as shown in Fig. 3. The comparison among previous models UB1997 [22], KB2005 [23], and our model K2014 is shown in Table 2 (only in the first seven rounds because from the eighth round, our network becomes too big to be presented here). Since pathways can be generated endlessly if reactions are still possible to happen according to the reaction rules, we can set *round* in the for loop and get a network of the size we need. At the same time, we can also operate the network generated by removing glycans that do not exist in the Golgi apparatus using the *pathway* class.

**Table 2:** A comparison among the reaction networks generated by previous models UB1997, KB2005, and our model K2014 (First 7 rounds)

Model	UB 1997	KB2005	K2014
Number of enzymes	8	11	22
Number of structures	4	14	31
Number of reactions	12	26	64

## Discussion

As can be seen from Table 2, our model generates a much more complex glycosylation reaction network. Here we consider one horizontal step as one *round*. Take round 7 as an example. In the model UB1997 [22] where only eight enzymes are considered, there are only four glycan structures in the network. Three monosaccharides are operated in this network: mannose, *N*-acetylglucosamine, and galactose. While in 2005, 11 enzymes are involved in the model KB2005 and 14 structures can be shown in round 7. In this model, the network shows the glycosylation process containing five monosaccharides being added to or cut from the visual glycan structures. However, in reality, the structures of glycan can be much more complicated, and more monosaccharides can be added to a glycan during the glycosylation process regarding enzyme specificity.

Concerning the fact that more enzyme reaction rules have been updated by biologists, we take the most up-to-date reaction rules into concern and involve them all in the construction of a glycosylation reaction network. From previous research [28–46], we know that all of these enzymes make contributions to the glycosylation process and should therefore be included into the construction of a glycosylation reaction network. Thus, the model K2014 is built. There are many more glycan structures than we have space to show here. As can be seen from Fig. 3, 31 glycan structures have been generated by the catalysis of these 22 enzymes (totally 27 enzyme reaction rules). Note that it provides a more complete understanding of the glycosylation process. The enzymatic rules collected from databases and literature show that these enzymes exist in cells and are involved in the glycosylation process. Concerning that our network includes more enzymes than the original GNAT and the model KB2005 [23], it is closer to reality. Thus, our model provides a result that better represents the underlying glycosylation process.

## Conclusions

In this work, we extend the model KB2005 [23] with application of the toolbox GNAT to construct a relatively larger *N*-linked glycosylation network. While GNAT only involves the presence of 9 enzymes, we include 22 enzymes (totally 27 enzyme reaction rules) whose information is collected from the literature, glycosylation-specific databases such as GlycomeDB [16], the Consortium for Functional Glycomics (CFG) website [25], KEGG Glycan and ggdb. Our framework K2014 can automatically construct a relatively larger glycosylation reaction network using 27 streamlined enzyme reaction rules. Accordingly, this framework advances the study of the *in silico* cell processes and potentially has significant benefits.

With this glycosylation network and related algorithms, a variety of network analysis strategies can be implemented to analyze the components of the overall glycosylation network. With our framework K2014, it is possible to analyze conventional biochemical or mass spectrometry-based experimental data quantitatively in a more realistic and practical way. Examples include, but are not limited to, a dynamic mathematical model for monoclonal antibodies (mAbs) glycosylation to estimate unknown enzymes and transport proteins (TPs) concentration profile parameters [47], a framework to quantitatively understand how changes in enzymes activities affect the profile of glycan structures produced in the biosynthesis process [24], and the simulations on *N*-glycan processing in accessing whether a homogeneous glycan profile can be created through metabolic engineering [48]. Future research can be conducted on the above-related issues using both conventional biochemical resources and high-throughput MS experimental data.
